# Impacts of Spatial Components on Outdoor Thermal Comfort in Traditional Linpan Settlements

**DOI:** 10.3390/ijerph19116421

**Published:** 2022-05-25

**Authors:** Lili Zhang, Haoru Liu, Dong Wei, Fei Liu, Yanru Li, Haolin Li, Zhuojun Dong, Jingyue Cheng, Lei Tian, Guomin Zhang, Long Shi

**Affiliations:** 1 College of Architecture and Urban-Rural Planning, Sichuan Agricultural University, Chengdu 611830, China; liuhaoru@stu.sicau.edu.cn (H.L.); s20172805@stu.sicau.edu.cn (D.W.); 79013@sicau.edu.cn (F.L.); liyanru@sicau.edu.cn (Y.L.); 2020225001@stu.sicau.edu.cn (H.L.); 2020325009@stu.sicau.cn (Z.D.); chengjingyue@stu.sicau.cn (J.C.); tianlei@stu.sicau.edu.cn (L.T.); 2 Civil and Infrastructure Engineering Discipline, School of Engineering, RMIT University, Melbourne, VIC 3000, Australia; kevin.zhang@rmit.edu.au (G.Z.); long.shi@rmit.edu.au (L.S.)

**Keywords:** outdoor thermal comfort, space composition, traditional settlement, ENVI-met, orthogonal test, Linpan

## Abstract

Traditional settlements have received increasing attention because of China’s rural revitalization. Traditional settlements with excellent thermal comfort in rural areas can attract urban residents, so it is vital to explore the thermal comfort of traditional settlements. For this paper, we studied Linpan settlements, which are scattered traditional settlements that are mainly composed of buildings and trees. Firstly, we visually interpreted Linpan settlements by ArcGIS. A total of 1194 Linpan settlements were classified to obtain the spatial components. The statistical results of Linpan were used in the subsequent experimental design. Then ENVI-met was used to simulate 25 different spatial forms of Linpan obtained by statistical results and orthogonal experiment to explore the most comfortable Linpan layout. The results showed the following: (1) Linpan could improve thermal comfort in both winter and summer. Adjusting the spatial arrangement could maximally increase the mean physiological equivalent temperature (PET) of the whole Linpan area by 1.03 °C in winter and reduce it by 3.02 °C in the summer. (2) At different time points, the influence of different space factors on thermal comfort was also different. The overall significance of each factor on thermal comfort was addressed as follows: vegetation coverage (highly significant) > building number (highly significant) > building form (highly significant) > vegetation distribution (significant), but the building distribution was not significant. (3) The best spatial arrangement scheme was high vegetation coverage, a large number of buildings, tri-courtyard buildings, surrounding vegetation distribution, and surrounding building distribution. The innovation of this paper lies in introduced thermal comfort into the traditional Linpan settlement, extracted spatial features of buildings and vegetation by visual interpretation combined with GIS software, and the fact that we conducted the experimental design of microclimate and thermal comfort based on spatial features. The research results can guide the outdoor thermal environment renewal design of Linpan and other traditional settlements.

## 1. Introduction

**Figure 1:**
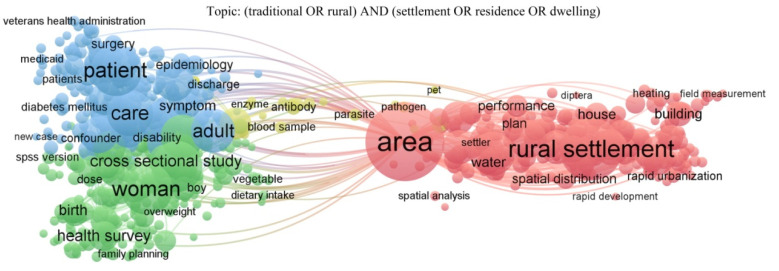
Analysis diagram of the literature on traditional or rural settlements in the past decade.

After China’s rural revitalization, ecological livability has become a guarantee of rural development, advocating that rural areas should protect the original ecosystem and achieve harmony between man and nature [1]. People have valued some traditional settlements, but the living environment of many traditional settlements is in urgent need of restoration and renewal. With the aggravation of global climate change, a green low-carbon lifestyle and the concept of harmonious coexistence between human beings and nature have gradually gained popular support [2,3]. In recent years, many residents are far away from the city and have begun to pay attention to the outdoor environment in rural areas, so it is imperative to improve and update the outdoor environment of traditional rural settlements. Figure 1 shows a co-occurrence analysis of nearly 3000 articles on rural or traditional settlement in the past decade. The focus of relevant research is mainly on patients (health), women (families), buildings, and settlements (spatial structure). Optimizing the ecological space for production and living, continuously improving the living environment, and building beautiful and livable villages are important strategies for China to promote rural revitalization comprehensively. It is imperative to improve and update the outdoor environment, such as the outdoor thermal comfort of the traditional settlement environment.

**Figure 2:**
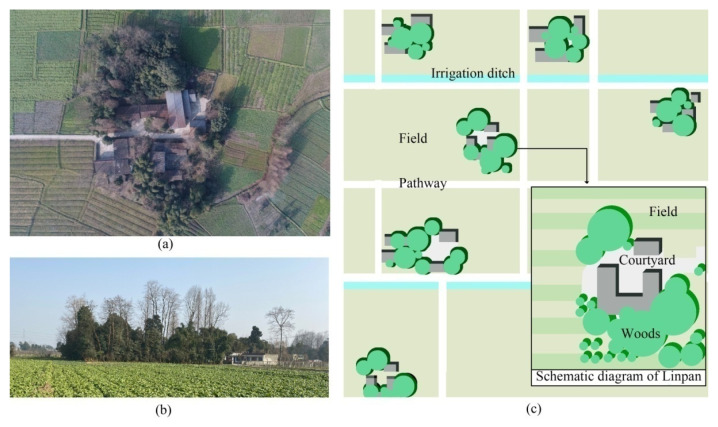
Real scene and structure diagram of Linpan in Chengdu Plain: (**a**) top view of a piece of Linpan, (**b**) side views of a piece of Linpan, and (**c**) distribution pattern and internal structure of Linpan.

Linpan refers to scattered traditional Chinese settlements distributed on the Chengdu Plain in Southwestern China. Its protection and restoration are among the key projects in response to rural revitalization in Chengdu. Linpan is dominated by buildings and trees, surrounded by fields for farming, such as green islands, which are good for regulating outdoor microclimate and thermal comfort. Linpan means “disk-like forest” in Chinese, and the total number of Linpan settlements in Chengdu Plain is about 200,000. Figure 2a,b shows the top and side view of a typical Linpan in Chengdu Plain. From the perspective of landscape science, the matrix of Chengdu Plain is vast fields, the corridors are field roads or ditches, and the patches are towns and tens of thousands of Linpan settlements. Woodlands, houses, courtyards, surrounding ditches, and fields are usually the basic elements of each traditional Linpan settlement [4,5]. The plane shape of Linpan can be divided into group and strip shapes [6]. Linpan is unusually dispersed due to geographical, historical, and cultural reasons [7]. Several to several hundred family members live in two- or three-story buildings in each Linpan [8]. Most of the buildings are built by villagers themselves, and almost every Linpan has a large number of planted trees. Clans inhabit most Linpan families; with the addition of population at home and the separation of brothers, Linpan will gradually develop and expand over time. The periphery of Linpan main body is generally cultivated land, which is closely combined with households, and the farming radius of Linpan is generally 100–200 m. Figure 2c is a schematic diagram of the spatial distribution and structure of the neighboring settlements, which can clearly show the spatial structure, including the green islands of Linpan. After the idea of rural revitalization, more attention has been paid to the protection and restoration of Linpan settlements in Chengdu, and many researchers have also conducted many studies on Linpan.

At present, the research about Linpan mainly focuses on structural characteristics, values, and protection. In terms of structural characteristics, Wan et al. [6,9] and Li et al. [5] studied the spatial layout structure and optimization of Linpan; Liu et al. [10] studied the composition and distribution of common plants of Linpan. In terms of Linpan values, the ecological [11,12], cultural [2], and aesthetic [13] values of Linpan have been studied by scholars. In terms of microclimate, Liu et al. [14] and Zong et al. [15,16] proved that Linpan could adjust outdoor microclimate in both winter and summer by field measurement. Only field measurements were used to study the microclimate of Linpan, and there is no measurement on different types or spatial components of Linpan; thus, this may affect the accuracy of the results. Few studies have put forward suggestions on the conservation of Linpan through quantitative analysis. Research on the coupling of space composition and outdoor environment mainly focuses on urban areas, and there are very few on the countryside [17].

Spatial elements such as vegetation, buildings, and water bodies can change the thermal comfort mainly by adjusting microclimate indexes such as temperature, humidity, wind speed, and solar radiation [18,19]. Green plants can positively affect the microclimate because the canopy of trees blocks and absorbs a large amount of solar radiation. In addition, the transpiration of vegetation significantly reduces the temperature of leaves and surrounding air [20], and trees can also guide airflow and reduce wind speed [21]. Different distribution and types of vegetation will affect outdoor microclimate and thermal comfort [22,23]. In addition, vegetation height, canopy width, and application location also significantly affect outdoor microclimate and thermal comfort. Some scholars pointed out that planting trees in front of a house on a sunny side is not the best way to shade from the sun [24,25], the thermal comfort of scattered trees is superior to centralized trees in both winter and summer, and it is recommended to plant trees with large crown [26]. On the other hand, although trees can shade and cool down in summer, they can reduce thermal comfort in cold regions or seasons [26,27]. Another spatial component showing an important influence on microclimate is buildings, including their distribution, density, aspect ratio and surface albedo, which can change the microclimate by directing or blocking air flows, increasing or blocking solar radiation. Generally, dense buildings can alleviate the thermal comfort in summer [28], but some studies have pointed out that the cooling of dense building layouts is not suitable for all cases and may also block the ventilation in summer [29,30]. Some scholars have discussed the influence of different sky view factors on the outdoor thermal environment and thermal comfort, pointing out that space with lower sky view factors would suffer from longer cold and uncomfortable period than other spaces in winter, although it showed lower heat condition in summer [31]. To sum up, the arrangement of buildings and vegetation should consider the characteristics of the macro-climate zone and the specific environmental conditions of the city, so as to balance the thermal comfort in winter and summer. Table 1 shows some residential outdoor microclimate references about space composition in recent years. As can be seen from the table, the spatial components that affect outdoor thermal comfort mainly include vegetation distribution, vegetation coverage, vegetation type, building distribution, and building coverage. The above research objects are mainly concentrated in cities.

Studies on the coupling of spatial composition and outdoor environment are mainly concentrated in urban areas, and the research results are far from supporting the development of rural human settlements. There are great differences in the spatial composition of different Linpan. However, only field measurements are used to study the microclimate of Linpan, and there is still a lack of research on the microclimate of different types of Linpan. Therefore, many experiments and numerical simulations about Linpan and outdoor thermal comfort have been carried out. This study aimed to achieve the following:

- Characterize the spatial composition and distribution of Linpan;
- Identify the optimal combination of Linpan and its high-impact variables;
- Prioritize the design of traditional settlements to improve outdoor thermal comfort.

## 2. Methodology

### 2.1. Site Selection and Data Statistics

**Figure 3:**
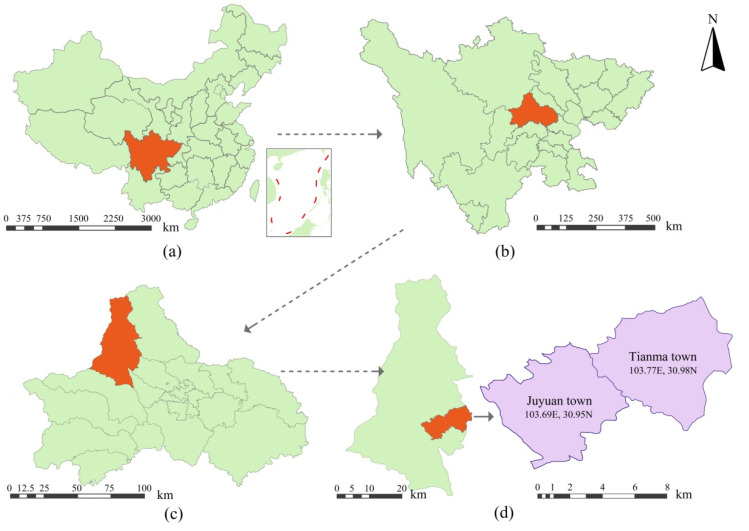
(**a**) Location of Sichuan Province in China. (**b**) Location of Chengdu city in Sichuan Province. (**c**) Location of Dujiangyan city in Chengdu city. (**d**) Location of Juyuan Town and Tianma Town in Dujiangyan city.

Dujiangyan city, in the west of Chengdu Plain, has a large density of Linpan settlements, with an average spacing of about 200 m [2]. Within the city limits of Dujiangyan, Tianma Town and Juyuan Town are the essence irrigation areas of Dujiangyan, with convenient transportation, a dense water network, few hills, and a large number of typical traditional Linpan settlements. Therefore, Linpan settlements in Juyuan Town and Tianma Town of Dujiangyan city were selected as the research objects. Figure 3 shows the locations of Juyuan Town and Tianma Town in Dujiangyan city.

Google Earth satellite images and ArcGIS were used to visually interpret the Linpan in Juyuan Town and Tianma Town of Dujiangyan city. In the 20-level high-definition satellite images, the outlines of buildings and woodland could be accurately identified. The main body of the Linpan is the part surrounded by the surrounding fields, composed of buildings and woodland to form an island in the areas. Therefore, the main body of the Linpan can be accurately recognized in the satellite images. Although many studies on the spatial components of Linpan can be found in recent years [5,6,9], this paper did not use the statistical data from these studies. The reasons are given as follows: First, the spatial data we needed include the number, density, spacing, distribution mode, and specific form of buildings, as well as the common types, coverage, and distribution of vegetation in Linpan, but these specific data are difficult to be obtained in the other references. Secondly, different scholars’ research was based on different cities, towns, and places, and the classification methods of Linpan species were also different. Therefore, the goal of interpretation was to extract the outlines of buildings, vegetation, and the main bodies of Linpan (composed of buildings, trees, and open spaces) and draw these contours into polygons for statistics.

### 2.2. Field Measurement

**Figure 4:**
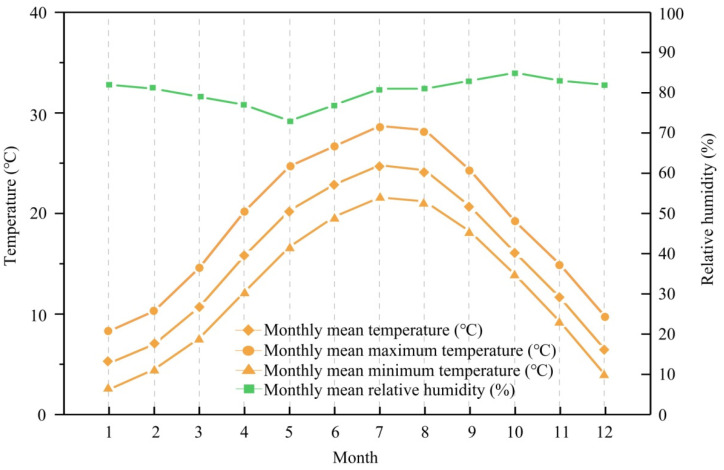
Map of historical meteorological data of Dujiangyan city.

**Figure 5:**
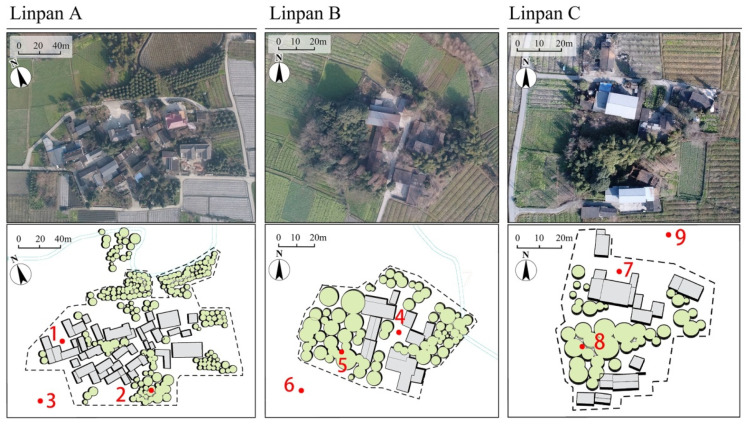
Measuring points distribution of Linpan A, B, and C.

According to historical meteorological data, the warmest month in Dujiangyan city is July, while the coldest month is January. Figure 4 shows the historical meteorological data of Dujiangyan city for 30 years [35]. The Linpan settlements in Juyuan Town and Tianma Town of Dujiangyan city were measured on consecutive sunny days of the coldest month in winter (January) and the hottest month in summer (July). Three adjacent Linpan were selected as the measured objects in winter and summer. A certain distance separated the three Linpan, and the vegetation distribution was different. Table 2 shows the area and internal composition of the three Linpan. Since the main body of Linpan is composed of buildings, woodlands, and open spaces and surrounded by surrounding fields, we arranged measuring points in different locations as a comparison. We arranged 9 measuring points in these three Linpan, with points 1, 4, and 7 in front of the open space of the buildings; points 2, 5, and 8 in the center of the woodlands; and the rest in the surrounding fields. Figure 5 shows the location of the selected measured stand and the measured point distribution. In winter, the measured time was three days on 14, 15, 18 January 2021, and the measurement time at each point was 24 h. In summer, the measured time was selected from 11–13 July. The measured data included the changes of air temperature and relative humidity at different points in a day. The test instrument TD-JTR08 (measurement range = −40 and 85 °C, 0–100%; accuracy = ±0.3 °C, ±1.5%; resolution ratio = 0.1% °C, 0.1%RH) was fixed at the height of 1.5 m above the ground and recorded data every 10 min, compliant with ASHRAE standard 55-2017.

In addition, the shape, size, building height, vegetation species, and distribution of each measured stand were measured and recorded, and these data were used to build models. As for the vegetation species in Linpan, we recorded the typical vegetation, as can be seen in Table 2.

### 2.3. Simulation Verification

In terms of model selection, ENVI-met [36] was used to model the Linpan and simulate the outdoor microclimate and thermal comfort. ENVI-met software is often used to simulate the influence of trees [26,27], water [37], surface materials [38], building layout [39,40], etc., and it has been verified by researchers from different countries in multiple climate regions around the world [41].

The applicability of the model in Linpan settlements should be verified. We constructed the Linpan A, B, and C model, and the related parameters are shown in Table 3. According to the tree characteristics in this table and the high leaf area density of vegetation, similar 3D trees were selected in the database (*Robinia pseudoacacia*, *Acer platanoides*, and cylindrical default trees). Due to the low height of the main body of models, isometric mesh was used in the vertical direction, and the lowest cell was split into five subcells. To ensure the stability of the model, 5 nested meshes were set at the model boundary to prevent the influence of boundary conditions [42]. The simulation time is the same as the measured date, and the time of each model was set to 30 h, while the data of the first 6 h of the initial simulation were abandoned. The weather and soil data came from hourly data provided by the Dujiangyan weather station [43]. In ENVI-met calibration, comparing temperature and relative humidity between simulated and measured values is a general method [27,33,44]. In this paper, hourly temperature and humidity values of 1.5 m height at the same position in the model are extracted and compared with the measured hourly average value.

After the ENVI-met simulation was completed, simulated air temperature (Ta) and relative humidity (RH) values were extracted from each point at the height of 1.5 m and compared with field measurements. Willmott [45] suggested the use of root mean square error (RMSE) and consistency index (d) to compare the measured and simulated results. The relevant calculation formula is as follows:

(1) $$\mathrm{RMSE}=\sqrt{\frac{1}{n}\sum_{i=1}^{n}{\left(P_{i}-O_{i}\right)}^{2}}$$

(2) $$d=1-\frac{\sum_{i=1}^{n}{\left(P_{i}-O_{i}\right)}^{2}}{\sum_{i=1}^{n}{\left(\left|{P^{\prime }}_{i}\right|+\left|{O^{\prime }}_{i}\right|\right)}^{2}}$$

where *P_i_* is the simulated value; *O_i_* is the measured value; *n* is the total number of samples; and *P′_i_* = *P_i_* − $\bar{O}$, *O′_i_* = *O_i_* − $\bar{O}$, and $\bar{O}$ are the average values of the measured values. When the RMSE value is as small as possible and *d* is greater than 0.7, the two sets of data can be considered related [33]. Tsoka et al. [41] mentioned that the acceptable RMSE of temperature should be less than 4.30 °C, and the RMSE of relative humidity should be less than 10.2% in their review. Table 4 shows RMSE and d-values between simulated and measured values of Linpan in winter and summer. The RMSE value of temperature and humidity in this paper do not exceed the critical value. We also found that the simulation results underestimated the daytime temperature in winter and summer and underestimated the fluctuation of temperature and humidity. The change was not as obvious as measured results. Previous studies also observed similar phenomena [25,28,46]. In summary, it can be seen that both the simulated and measured values in this paper are within an acceptable range, and it is feasible to use ENVI-met to simulate the microclimate and thermal comfort in Linpan settlements.

### 2.4. Thermal Comfort Index

Thermal comfort is a subjective thermal response of the human body to thermal sensation and a state of expressing satisfaction with the current thermal environment. Thermal comfort is often affected by physical environmental conditions, human activity, clothing, and psychological [47]. This paper selected physiological equivalent temperature (PET) to evaluate outdoor thermal comfort. PET applies to indoor and outdoor conditions, and PET has been formally used in an outdoor environment by the German Meteorological Office and is widely used in outdoor thermal comfort assessment [48]. The metabolic rate and clothing thermal resistance were set to default values of 80 W and 0.9 clo in ENVI-met, respectively. According to the *Thermal Design of Civil Buildings Code*, Chengdu belongs to hot summer and cold winter areas [49]. This paper used modified PET, which is suitable for hot summer and cold winter areas, as the evaluation index of thermal comfort [50]. Table 5 shows the modified PET classification for areas with hot summers and cold winters.

## 3. Spatial Composition Analysis and Design

### 3.1. Results of Spatial Composition Analysis

**Figure 6:**
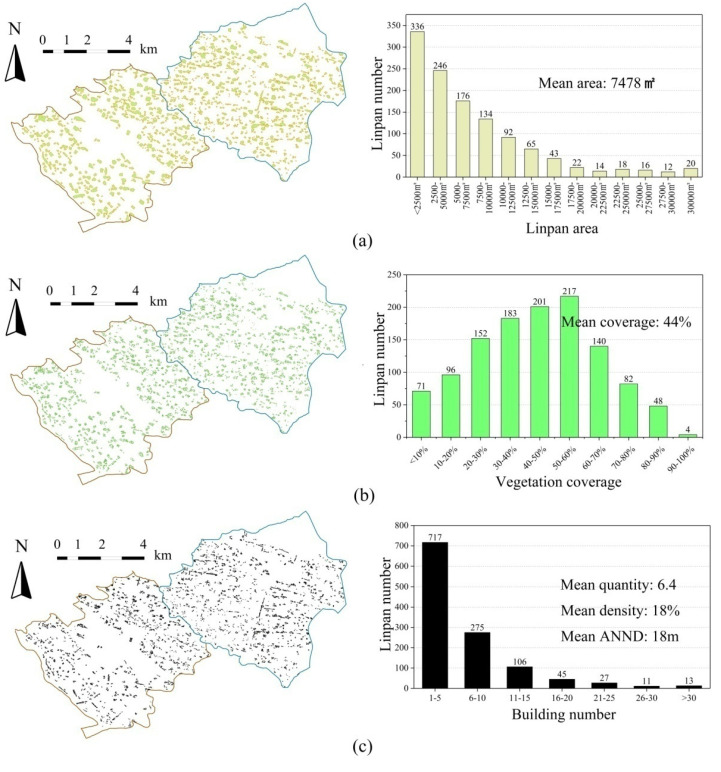
Linpan area, vegetation coverage, building number, density, ANND statistical results. (**a**) Linpan main body area statistics; (**b**) Linpan vegetation coverage statistics; (**c**) The statistics of building number, mean building density and mean ANND in 1194 Linpan.

**Figure 7:**
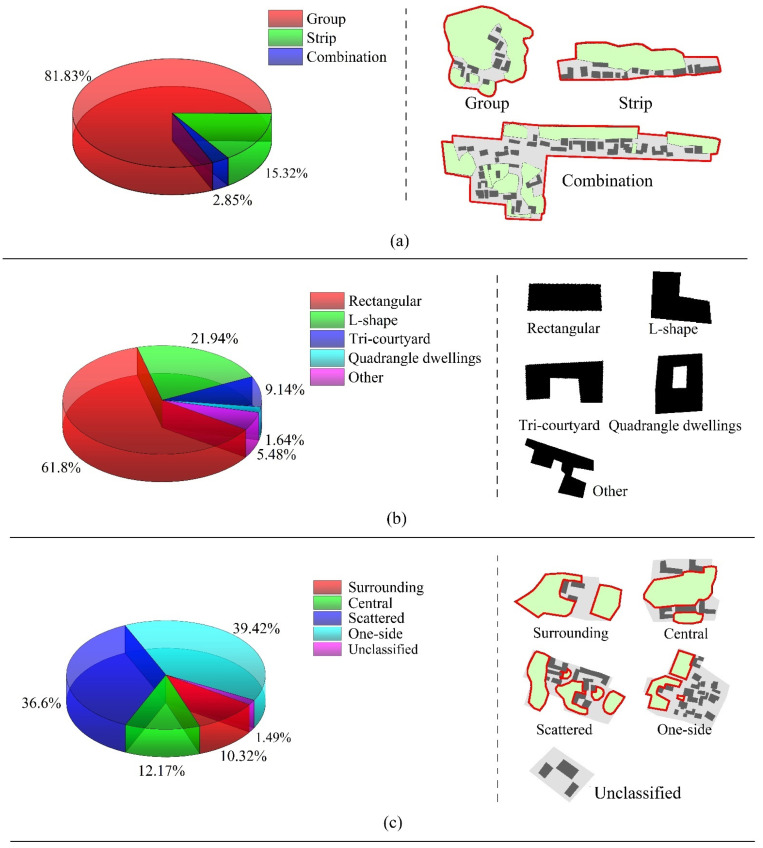
Classification results in main body shape, architectural form, and vegetation distribution: (**a**) pie chart of Linpan shapes and schematic diagram, (**b**) pie chart of building form and schematic diagram; and (**c**) pie chart of vegetation distribution and schematic diagram.

A total of 1194 Linpan, 7980 buildings, and 1257 pieces of woodlands were extracted from Juyuan and Tianma town, and the composition of each Linpan was analyzed. Figure 6 shows the main body area of the Linpan, the specific situation of the internal building density and vegetation coverage. The average area of the main body of the Linpan is 7478 m^2^, and the largest number of Linpan are those smaller than 2500 m^2^. With the increase of the main body area of the Linpan, the number of Linpan gradually decreases, as shown in Figure 6a. The average vegetation coverage rate was 44%, and the number of Linpan with a vegetation coverage rate of 50–60% was the largest, as shown in Figure 6b. Linpan containing 1–5 buildings account for the largest proportion, with an average density of 18%, as shown in Figure 6c. In addition, ArcGIS extracted the centroid of each building surface and calculated the average nearest neighbor distance (ANND) of each building inside each Linpan. The calculation method of the average closest neighbor distance is as follows:

(3) $$\mathrm{ANND}=\frac{\sum_{i=1}^{n}d_{i}}{n}$$

where *d_i_* is the shortest distance from one building centroid to another building centroid in a piece of Linpan, and n is the total number of all building centroids in a piece of Linpan. The results showed that the average ANND of all Linpan was 18 m, and the Linpan with an interval between 15 and 20 m was the most numerous, followed by those with an ANND between 21 and 25 m. Figure 7 shows the Linpan main body shape, building form, and vegetation distribution forms of classification results. The classification of the main body shape and architectural form of Linpan is common in reality, and we also referred to others’ classifications [2,6]. The buildings mainly face south. As shown in Figure 7a, Linpan main body shape in the majority with group shape. Rectangular buildings account for the highest proportion of interior buildings, followed by L-shaped ones (Figure 7b). The vegetation distribution pattern is mostly unilateral, followed by scattered, central, and surrounding, as shown in Figure 7c, as is consistent with the classification of Linpan vegetation by Zong et al. [15]. These statistical results were used in the later simulation experiment design of thermal comfort.

### 3.2. Orthogonal Experimental Design

In the grid area of 86 m × 86 m × 30 m, which was similar to the average area of 7478 square meters shown in Figure 6a, a square Linpan model was established for simulation. We set the cell size to 2 m × 2 m × 3 m and attached five nested grids to the model. Hourly meteorological parameters in winter and summer were input in accordance with the typical meteorological day data [51] of Chengdu in *Design standard for thermal environment of urban residential areas* and its draft for soliciting opinions. Selecting typical meteorological days for the simulation can fully reflect the microclimate change of traditional houses in the long-term meteorological change. The wind in winter was set at 0.9 m/s (22.5° direction) and in summer at 1.35 m/s (337.5° direction). Except for the model domain, plants, and meteorological conditions mentioned in this paragraph, other settings were the same as in Table 3.

**Figure 8:**
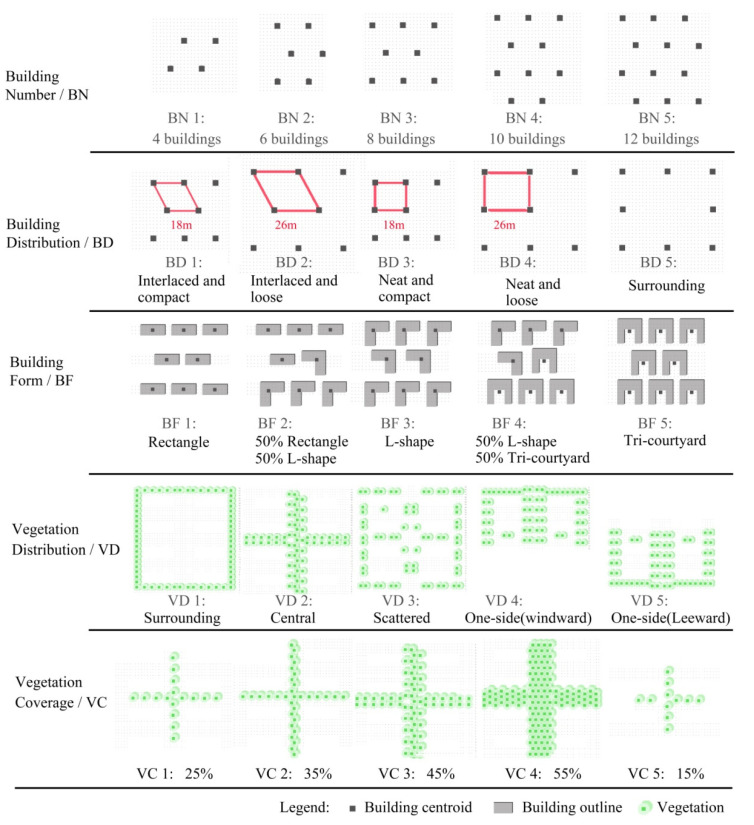
Experimental design levels for all factors.

**Figure 9:**
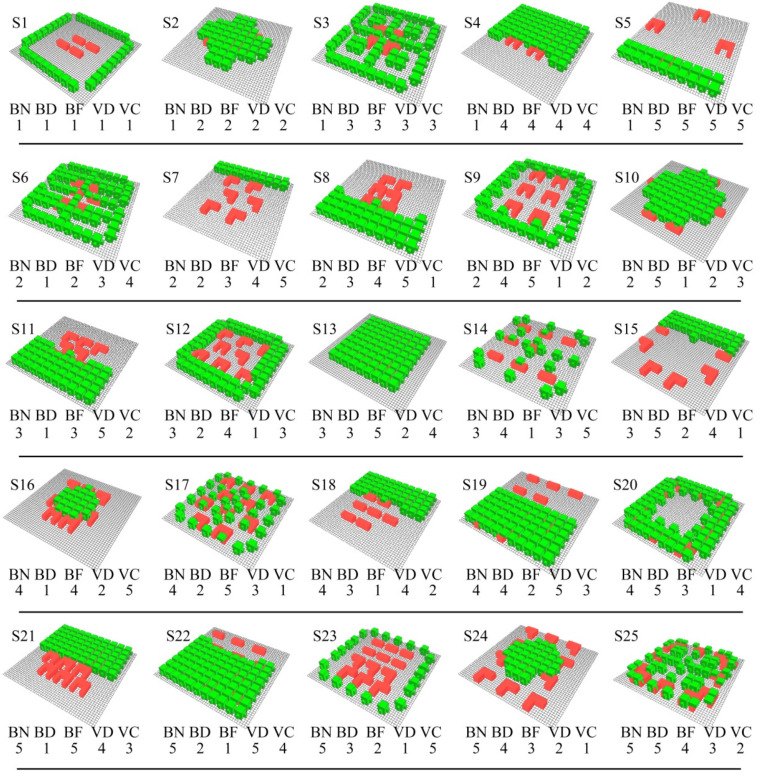
Schematic diagram of 25 experimental schemes.

To improve the efficiency of the experiment, the orthogonal experiment method was introduced in this paper for experimental design, which could significantly reduce the number of study cases. The purpose of the orthogonal experiment was to determine the importance of test factors and their influence on test indicators, the optimal combination of factors, and the quantitative relationship between indicators and factors [44]. L25 (5^6^) was selected as the orthogonal table of the experiment, which required 25 experiments. Each row in an orthogonal table represents an experimental scheme, and each column represents an experimental factor. Building number, building distribution, building form, vegetation distribution and vegetation coverage are shortened as BN, BD, BF, VD, and VC, respectively. The rightmost column is blank row, which is used as an error column (e). Five levels were designed in BN factor according to the average number and the high-frequency range shown in Figure 6c: 4, 6, 8, 10, and 12 buildings levels. Five levels were designed in BD factor according to ANND results in Section 3.1: interlaced and compact (ANND = 18 m), interlaced and loose (ANND = 26 m), neat and compact (ANND = 18 m), neat and loose (ANND = 26 m), and surrounding distribution. Three levels were designed in BF factor according to the results shown in Figure 7b: rectangular, L-shape, and tri-courtyard buildings. Five levels were designed in VD factor according to the results shown in Figure 7c: surrounding, central, scattered, one-side, and the prevailing wind in Chengdu is north wind in winter and summer, so that the one-side was divided into upwind and leeward levels. Five levels were designed in VC factor according to the average value 44% and the high-frequency range shown in Figure 6b: 25%, 35%, 45%, 55%, and 15% coverage rate of vegetation. We did not select BN level less than four buildings and VC level over 60% coverage because it was very difficult to design building distribution scheme with small BN and a vegetation distribution scheme with high-density VC. Figure 8 shows all of the levels and corresponding labels for each factor. To make the level number of each factor the same for the orthogonal experiment, two levels were added in BF factor: 50% rectangle/50% L-shape (BF2) and 50% L-shape/50% tri-courtyard (BF4), as can be seen in Figure 8. In terms of vegetation type, we selected the local common trees as the vegetation model (height, 9 m; width, 7 m). Figure 9 shows all 25 design schemes of the orthogonal table.

## 4. Results and Discussions

### 4.1. Results of ANOVA

In this part, we use the analysis of variance (ANOVA) to determine the influence of each factor on the PET. ANOVA can estimate the size of errors and accurately estimate the importance of the influence of various factors on experimental results. After the simulation of each scheme, we extracted the PET results of 11 h, from 08:00 to 18:00 (representing daytime hours for daily activities) for ANOVA. Generally, there are two methods to compare the results: one is to select one or several monitoring points in the area for comparison, and the other is to compare the average PET of all grids in the area. This paper selects the second method because there are many changes in buildings and vegetation in Linpan. The results of each scheme were obtained by calculating the average PET of the simulative domain of each scheme. We compared the PET results of 25 schemes and found that adjusting the spatial arrangement could maximally increase the average PET of simulative domain by 1.03 °C in winter and reduce 3.02 °C in summer. Table A1 in Appendix A shows each scheme’s average PET simulation results. This shows that adjusting the spatial arrangement of Linpan has positive effects on improving outdoor thermal comfort. Table 6 shows the results of ANOVA on PET in winter and summer.

In the winter, vegetation coverage was the most significant factor, except for 09:00. Building number, building distribution, building form, and vegetation distribution had different influences at different time points in winter, as shown in Table 6. We conducted ANOVA on the average results in the daytime (dPET), and the results showed that the order of influence of various factors on dPET was vegetation coverage > building form in winter. Vegetation coverage was highly significant, building form was significant, and others had no significant influence on dPET. In the summer, vegetation coverage was also the most significant factor except 08:00, whereas building distribution was the least significant factor at any time. Other factors had different influences at different time points in summer. The results of dPET showed that vegetation coverage and building form had highly significant effects; building number had significant effects, while others were not. The order of influence was vegetation coverage > building form > building number in summer.

In general, the order of influence of factors on PET varies in different seasons and different time points; the most significant factor influencing PET was vegetation coverage in both the winter and summer.

### 4.2. Influence of Buildings on PET

**Figure 10:**
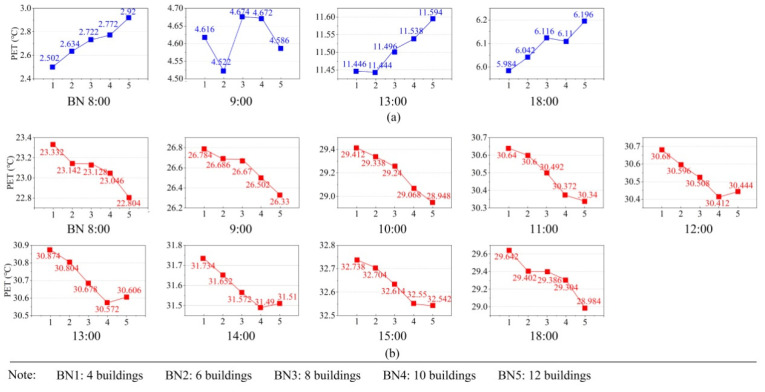
Effect diagrams of building number (BN) in winter and summer: (**a**) effect diagrams of BN in winter and (**b**) effect diagrams of BN in summer.

In this part, we found the optimal levels of building number, building distribution, and building form factors through single-factor analysis. The higher PET value in winter represents the more excellent level, while the lower PET value in summer represents the more excellent one. In terms of building number, the influence of five levels at different time points in winter and summer was calculated. Figure 10 shows the impact of building-number levels on PET. With the increase of building number in experimental Linpan, the PET values showed an upward trend overall, except for 09:00 in winter, as is shown in Figure 10a. On the other hand, with the increase of building number, the PET values showed a decreasing trend overall in summer, as is shown in Figure 10b. Regardless of winter or summer, four or six buildings with a small BN were most unfavorable to thermal comfort, whereas buildings with a high coverage rate were most favorable to thermal comfort. BN5 (12 buildings) was the optimal level of building number in both winter and summer. Increasing the number of buildings helped to improve thermal comfort in both winter and summer, possibly because more buildings provided more shades in the summer [30,40] and effectively protected from cold winds in the winter. There was no significant regularity in BN at 09:00 in the winter, and this may be caused by ground warming and building shadow cooling. The PET of BN5 was higher than that of BN4 from 12:00 to 14:00 in the summer, which may be caused by the high solar angle at noon, the reduction of shadows, and the building obstructing ventilation.

**Figure 11:**
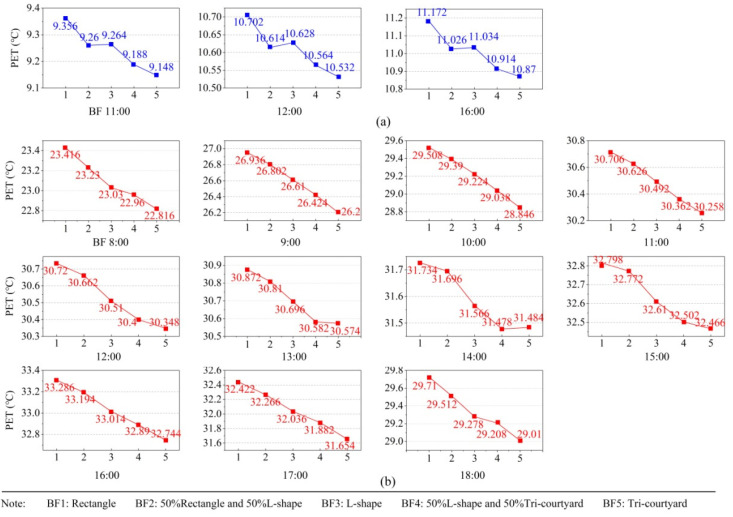
Effect diagrams of building form (BF) in summer: (**a**) effect diagrams of BF in winter and (**b**) effect diagrams of BF in summer.

Another factor closely related to building density is the building form. Figure 11 shows the effect of building form levels in summer. It can be clearly seen that, with the increase of building complexity (from BF1 to BF5), the PET in both winter and summer presents a downward trend overall. BF1 (rectangle) was the optimal level of building form in winter, whereas BF5 (tri-courtyard) was optimal in summer. Increasing the number and complexity of buildings both increased the density of Linpan, but we found that increasing the building number created better thermal comfort in winter, while increasing building complexity did the opposite. The reason for the difference may be that a complex building form could provide more shadow areas to lower ground temperature, but the block wind effect was not as good as increasing BN. A study [29] pointed out that building density did not significantly impact PET of street canyons in summer. The reason for the difference in this study may be that the range of building density in Linpan was larger, and Linpan was different from the street canyon. Middel et al. [28] and Galal et al. [30] proved that dense low-rise layouts of buildings were conducive to daytime cooling, and we found similar results.

**Figure 12:**
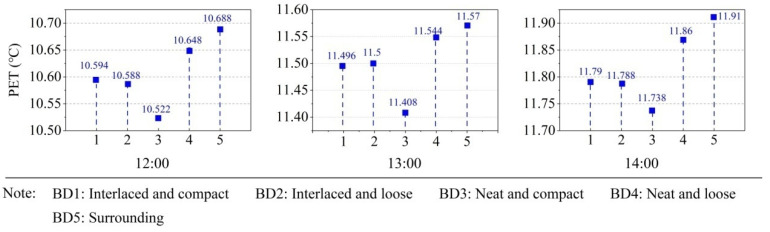
Effect diagrams of building distribution (BD) in winter.

In terms of the building distribution, significance was observed during 12:00–14:00 in the winter. Figure 12 shows the effect of building distribution levels in the winter. It could be seen that the BD5 (surrounding buildings) had the highest PET values, while BD3 (neat and compact) had the lowest PET value during 12:00–14:00. The reason may be that the surrounding buildings provide a larger area of non-shadow, allowing the ground to receive more solar radiation at noon.

In general, the 12-buildings level was the most favorable level for thermal comfort in winter and summer. The rectangle building level was the most favorable level in the winter, while the tri-courtyard level was the most favorable in the summer. The surrounding building level was the most beneficial level at noon in winter. Increasing building density was beneficial for summer thermal comfort, but increasing building number and building complexity in winter had a different effect.

### 4.3. Influence of Vegetation on PET

**Figure 13:**
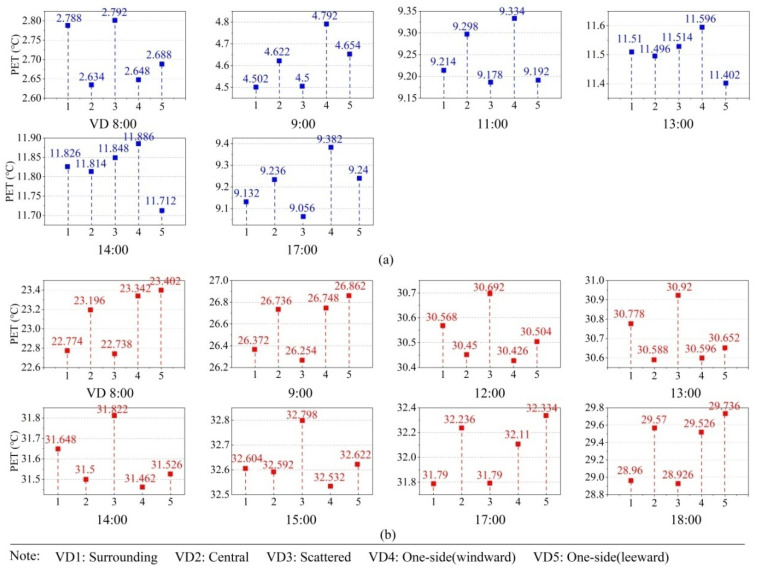
Effect diagrams of VD (vegetation distribution) in winter and summer: (**a**) effect diagrams of VD in winter and (**b**) effect diagram of VD in summer.

In this part, we found the optimal levels of vegetation distribution and vegetation coverage factors through single-factor analysis. Figure 13 showed the effect of vegetation distribution levels in the winter and summer in terms of vegetation distribution. At 08:00 in winter, VD3 (scatted) was the most conducive to thermal comfort. VD4 (windward) had the highest PET values at 09:00, 11:00, 13:00, 14:00, and 17:00 in the winter. The reason may be that the windward vegetation blocked the cold wind in the winter and provided larger solar radiation areas than the scattered one. In summer, VD3 was the most conducive to cooling during 08:00–9:00 and 17:00–18:00, but it was the least conducive during 12:00–15:00. The reason may be that the scattered vegetation in summer reduces wind speed at midday, making it harder to carry heat away and creating more hot zones around trees. Zhang et al. [26] mentioned that scattered trees reduced wind speeds. VD4 was most conducive to cooling at 12:00, 14:00, and 15:00. Zhang et al. [26] also mentioned that scattered trees were good for winter and summer, but this paper found that the windward vegetation was more conducive to thermal comfort in small-scale areas in winter.

**Figure 14:**
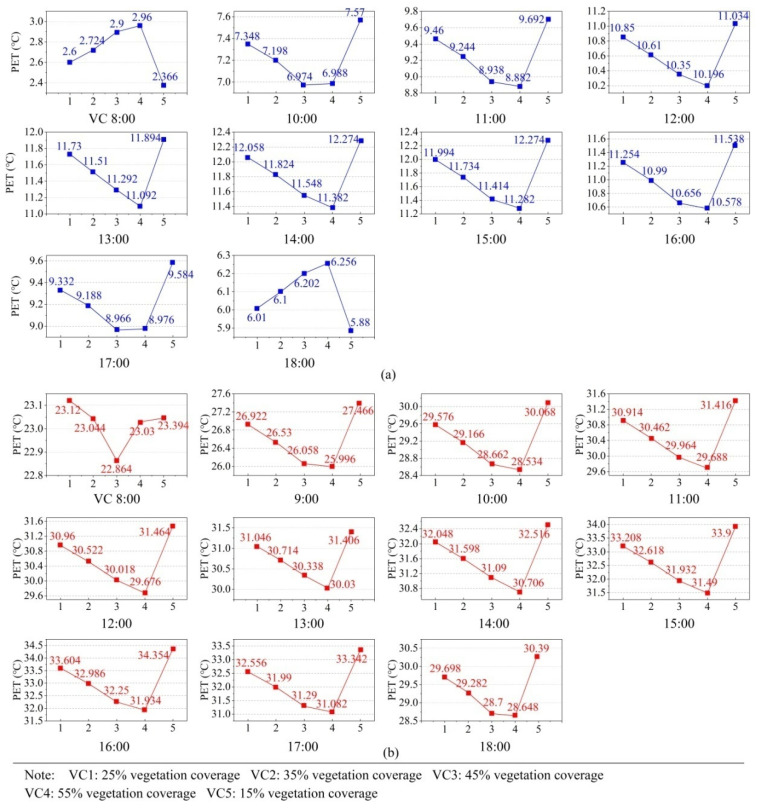
Effect diagrams of VC (vegetation coverage) in winter and summer: (**a**) effect diagrams of VC in winter and (**b**) effect diagrams of VC in summer.

In terms of the vegetation coverage factor, Figure 14 shows the effect of vegetation coverage levels in winter. As shown in this figure, with the increase of VC (from 15% to 55%), the PET showed an increasing trend at 08:00 and 18:00 in winter, while it showed a downward trend during 10:00–17:00. Solar radiation intensity had little influence at 08:00 and 18:00, and the higher vegetation coverage was, the more it could block the cold wind in winter. However, with the strengthening of solar radiation, the high-density vegetation reduced the absorption of solar radiation from the ground [26,27]. In summer, it is obvious that PET values decreased with the increase of vegetation coverage during 09:00–18:00. VC5 (15%) was the best level in winter, and VC4 (55%) in summer. The changing trend of the PET value at 08:00 was not significant because the temperature was not high enough. Wu et al. [25] and Wang et al. [46] also found similar results that trees had a better cooling effect on hot days.

In general, the windward distribution of vegetation was the most favorable level for thermal comfort in winter and summer. Overall, 15% vegetation coverage was the most favorable level in winter, while 55% vegetation coverage was the most favorable in summer. With the increase in vegetation coverage, Linpan produced better thermal comfort in summer but completely the opposite in winter.

### 4.4. Thermal Comfort Improvement Design Using Optimal Scheme

**Figure 15:**
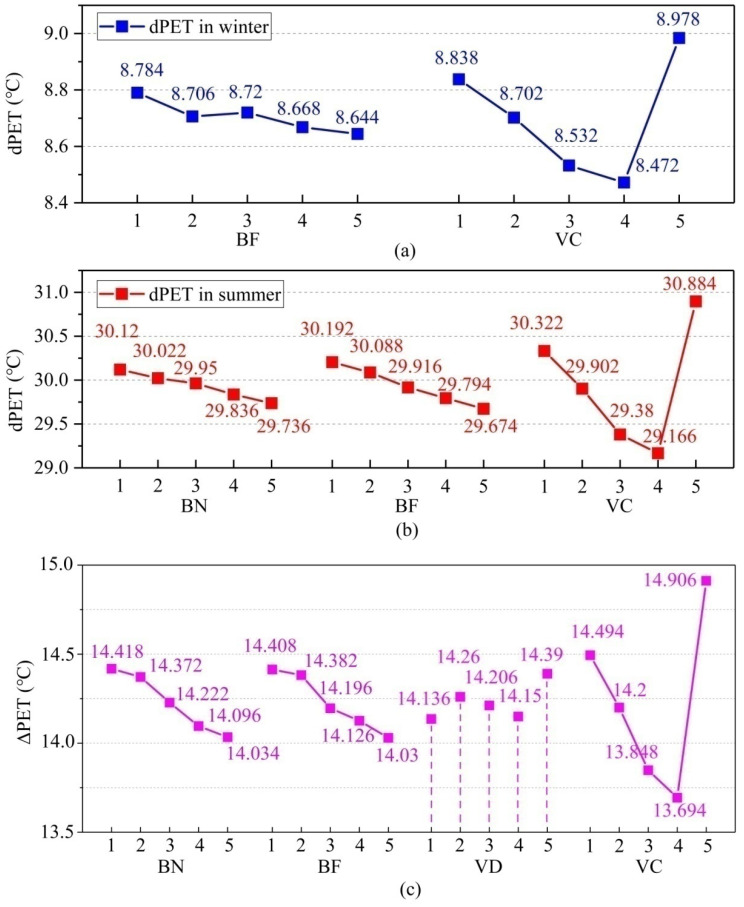
(**a**) Effect diagrams of significant factors on dPET in winter. (**b**) Effect diagrams of significant factors on dPET in summer. (**c**) The influence of factors and levels on ΔPET.

**Figure 16:**
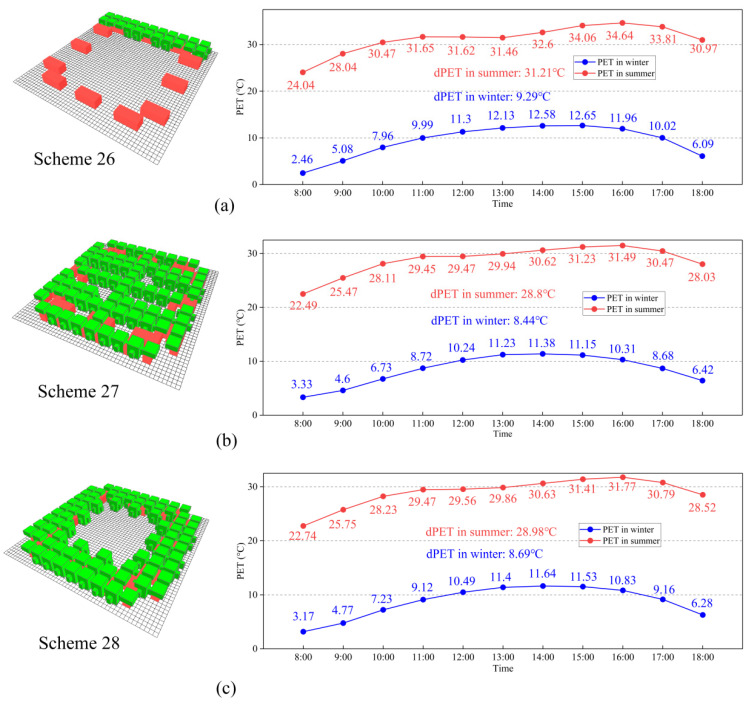
Hourly simulation results for Schemes 26, 27, and 28: (**a**) hourly simulation results for Scheme 26, (**b**) hourly simulation results for Scheme 27, and (**c**) hourly simulation results for Scheme 28.

In this part, we designed optimal schemes for different seasons and integrated the PET values of winter and summer to obtain the most suitable spatial arrangement scheme of Linpan. In Table 6, we found that BF and VC factors had significant effects on dPET in winter, while BN, BF, and VC factors had significant effects in summer. Figure 15 shows the effects of each significant factor on dPET in winter and summer. According to this figure, BF1 and VC5 were the best levels in winter, while BN5, BF5, and VC4 were the best levels in summer. In winter, BN, BD, and VD had no significant effect on dPET, but we took BN5, BD5, and VD4 as the optimal levels according to the winter high-frequency optimal levels, as shown in Figure 10, Figure 12 and Figure 13. Therefore, the best scheme suitable for Linpan in winter was as follows: BN5, BD5, BF1, VD4, and VC5. We identified this scheme as Scheme 26. Similarly, in summer, the best scheme suitable for Linpan was as follows: BN5, BF5, VD3, and VC4, and we set BD to BD5 the same as in winter (Scheme 27). By simulating Schemes 26 and 27, it can be expected that Scheme 26 has the most comfortable PET value compared with options 1–25 in winter, and Scheme 27 has the most comfortable PET value in summer. Figure 16a,b shows the hourly simulation results for Schemes 26 and 27.

To find the optimal scheme and integrate the levels of winter and summer, we set new indices to study. According to Table 5, the PET range of 15–22 °C is comfortable. We set ΔdPET_S_ as the difference between the dPET in summer and 22 °C (ΔdPET_S_ = dPET – 22 °C), and we set ΔdPET_W_ as the difference between 15 °C and the dPET in winter (ΔdPET_W_ = 15 °C − dPET). The ΔPET value is taken as the sum of ΔdPET_W_ and ΔdPET_S_ values (ΔPET = ΔdPET_W_ +ΔdPET_S_) and used as an indicator to judge the optimal solution. The smaller the ΔPET is, the better the scheme is. The relevant values of each scheme are shown in Table 7.

We take ΔPET as new results for ANOVA and single-factor analysis. Table 8 shows the results of ANOVA on ΔPET, and it can be seen that factors except BD have significant influences. The order of influence is as follows: vegetation coverage > building number > building form > vegetation distribution. Figure 15c shows the influence of factors and levels on ΔPET. As can be seen from the figure, the optimal levels (the minimum level of ΔPET) are BN5, BF5, VD1, and VC4. For BD factors, although not significant for dPET or ΔPET, BD5 is the most suitable according to the optimal level of high frequency shown in Figure 12. Therefore, the optimal scheme for Linpan in winter and summer is as follows: 12 buildings, surrounding distribution buildings, tri-courtyard buildings, surrounding vegetation, and 55% coverage vegetation, and we identified this as Scheme 28. Figure 16c shows hourly simulation results for Scheme 28. This arrangement is beneficial to both winter and summer, providing sufficient solar radiation in winter and sufficient shadow area in summer.

**Figure 17:**
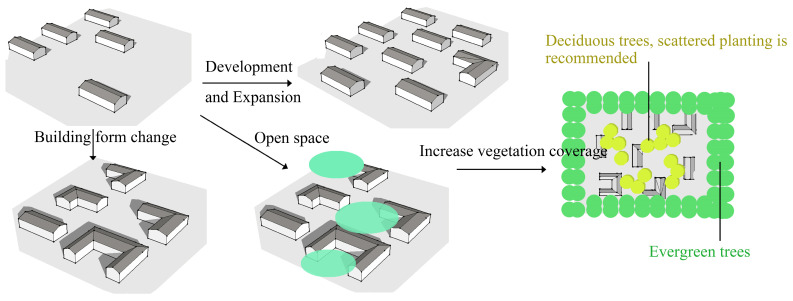
Suggestions for improving the thermal comfort of Linpan.

It is found that high-density and tri-courtyard buildings are beneficial to thermal comfort. Increasing vegetation coverage is beneficial to thermal comfort in the summer, but not in the winter. Therefore, we suggest planting high-density and surrounding vegetation, as with Scheme 28. It should be noted that Linpan-like Schemes 26 and 28 are rare in reality, as it is rare for villagers to build large open spaces inside Linpan. Therefore, we suggest the following: (1) Increase the density of buildings appropriately, expand the open space between buildings, and use the building form L-shape or Tri-courtyard. (2) Increase vegetation coverage, and plant native evergreen trees (camphor, nanmu phoebe, etc.) around Linpan. It is recommended to plant scattered deciduous trees (ginkgo, taxodium, etc.) inside Linpan, as shown in Figure 17.

## 5. Conclusions

This paper classified and counted 1194 Linpan by GIS software. ENVI-met was used to simulate 25 different spatial forms of Linpan obtained by statistical results and orthogonal experiment to explore the most comfortable layout in winter and summer. The following conclusions were obtained:

- The shapes of Linpan were mostly groups, and the internal traditional building forms were mainly rectangle, L-shape, and tri-courtyard, and the distribution modes of vegetation were mainly surrounding, central, scattered, and one-side layout.
- The Linpan settlements could produce a cooling effect in summer and a heating effect in winter. Adjusting the spatial arrangement of Linpan could maximally increase the mean PET of the whole space of Linpan by 1.03 °C in winter 16:00 and reduce by 3.02 °C in summer 16:00.
- In different seasons and different time points during the daytime, the significance of various factors on thermal comfort changed greatly. In winter, the order of factors’ influence was as follows: vegetation coverage (highly significant) > building form (significant). In summer, the order of influence was as follows: vegetation coverage (highly significant) > building form (highly significant) > building number (significant). Finally, we established the ΔPET, an index to evaluate thermal comfort in winter and summer. The influence of various factors on ΔPET was as follows: vegetation coverage (highly significant) > building number (highly significant) > building form (highly significant) > vegetation distribution (significant), and building distribution was not significant. The effect of vegetation coverage on outdoor thermal comfort was the most important.
- The best arrangement scheme in winter was as follows: large number of buildings, surrounding building distribution, rectangle buildings, windward vegetation distribution, and low coverage vegetation. In summer, it was as follows: large number of buildings, tri-courtyard buildings, scatted vegetation distribution, and high coverage vegetation. After integrating the results of winter and summer, we found that the best scheme to balance was as follows: large number of buildings, surrounding building distribution, tri-courtyard buildings, surrounding vegetation distribution, and high coverage vegetation.

Today, about 200,000 Linpan are distributed in the vast rural areas of Chengdu Plain, and many Linpan are facing reconstruction, repair, and reconstruction. We want to put forward relevant suggestions, such as increasing the building density and tri-courtyard buildings, widening the space between buildings as much as possible, increasing vegetation coverage, planting evergreen trees around Linpan, and planting scattered vegetation deciduous trees inside Linpan. After this, whether the experience of thermal comfort construction of Linpan can be applied to other traditional settlements is the question we will study next. We not only hope to provide guidance for the reconstruction and restoration of Linpan in Western Sichuan, but also hope to provide reference for the restoration of similar settlement structures and contribute to rural revitalization.

## Figures and Tables

**Table 1:** References of residential outdoor microclimate research about space composition in recent years.

Impact Elements	References	Methods	Season	Location	Thermal Comfort Index
Vegetation Distribution	[22]	FM, NS	Spring, Summer	Tianjin, China	PET, WBGT
	[25]	FM, NS	Summer	Beijing, China	-
	[23]	FM, NS	Summer	Berlin, Germany	PET
	[24]	FM, NS	Summer	Phoenix, AZ, USA	PET
	[26]	FM, NS	Summer, Winter	Wuhan, China	PET
Vegetation Coverage	[25]	FM, NS	Summer	Beijing, China	-
	[24]	FM, NS	Summer	Phoenix, AZ, USA	PET
	[32]	NS	Summer	Nanjing, China	PMV
	[27]	FM, NS	Summer, Winter	Tabriz, Iran	PET
	[33]	FM, NS	Summer	Guangzhou, China	-
	[29]	FM, NS	Summer	Bangkok, Thailand	PET
Vegetation Type	[22]	FM, NS	Spring, Summer	Tianjin, China	PET, WBGT
	[34]	FM	Summer	Beijing, China	DI
	[26]	FM, NS	Summer, Winter	Wuhan, China	PET
	[23]	FM, NS	Summer	Berlin, Germany	PET
	[27]	FM, NS	Summer, Winter	Tabriz, Iran	PET
Buildings Distribution	[28]	FM, NS	Summer	Phoenix, AZ, USA	-
	[32]	NS	Summer	Nanjing, China	PMV
	[30]	FM, NS	Summer	New Aswan, Egypt	PET
	[29]	FM, NS	Summer	Bangkok, Thailand	PET
Buildings Coverage	[28]	FM, NS	Summer	Phoenix, AZ, USA	-
	[32]	NS	Summer	Nanjing, China	PMV
	[29]	FM, NS	Summer	Bangkok, Thailand	PET

FM, field measurement; NS, numerical simulation; PET, physiological equivalent temperature; WBGT, wet bulb globe temperature; PMV, predicted mean vote; DI, Discomfort Index.

**Table 2:** The composition of Linpan A, B, and C.

		Linpan A	Linpan B	Linpan C
Vegetation	Area	3540 m^2^	3295 m^2^	1849 m^2^
	Area percentage	30.7%	68%	45.3%
	Distribution	Scattered	Surrounding	Central
	Vegetation Type	*Phoebe zhennan* S. Lee (height: 8–10 m; width: 6–7 m)		
		*Cinnamomum camphora* (L.) Presl. (height, 8–12 m; width, 6–7 m)		
		*Metasequoia glyptostroboides* (height, 15 m; width, 7–9 m)		
		*Ginkgo biloba* L. (height, 9–15 m; width, 7–9 m)		
Buildings	Area	2975 m^2^	712 m^2^	792 m^2^
	Area percentage	25.8%	14.7%	19.4%
Open space	Area	5016 m^2^	4844 m^2^	1441 m^2^
	Area percentage	43.5%	17.3%	35.3%

**Table 3:** ENVI-met simulation parameter setting.

Module	Related Parameters	Input Values
Model domain	Cell size	x = 2 m, y = 2 m, z = 3 m
	Cells number (x × y × z)	110 × 90 × 15 (Linpan A) 90 × 63 × 15 (Linpan B) 60 × 75 × 15 (Linpan C)
Meteorology	Air temperature (°C)	Hourly data from weather station (14–15 and 18 January 2021)
	Relative humidity (%)	Hourly data from weather station (11–13 July 2021)
	10 m height wind speed(m/s)	1.0 (January), 1.5 (July) (Data from weather station)
	Wind direction (°)	330 (January), 115 (July)
	2500 m height moisture content	7 g/kg *
	Cloud cover	0 *
	Reference roughness length	0.10
Plants	3D Plants	Table 3
Building	Roof albedo	0.3 *
	Wall albedo	0.2 *
Soil	Soil properties Soil relative humidity (%)	Loamy soil
		Winter: 80 (upper), 80 (middle and deep), 75 (bedrock) Summer: 70 (upper), 75 (middle and deep), 75 (bedrock)
	Soil temperature (°C)	Winter: 6 (upper), 6 (middle and deep), 8 (bedrock) Summer: 24 (upper), 24 (middle and deep), 24 (bedrock)

* The default value.

**Table 4:** RMSE and d-values about air temperature and relative humidity.

	RMSE		d	
	Winter	Summer	Winter	Summer
Air temperature	2.288	1.366	0.928	0.934
Relative humidity	9.879	6.489	0.938	0.887

**Table 5:** Modified PET classification for areas with hot summers and cold winters.

PET (°C)	Thermal Sensation	Thermal Stress Level
PET > 46	Very hot	Extreme heat stress
38 < PET ≤ 46	Hot	Strong heat stress
30 < PET ≤ 38	Warm	Moderate heat stress
22 < PET ≤ 30	Slightly warm	Slight heat stress
15 < PET ≤ 22	Neutral (Comfortable)	No thermal stress
7 < PET ≤ 15	Slightly cool	Slight cold stress
−1 < PET≤ 7	Cool	Moderate cold stress
−8 < PET ≤ −1	Cold	Strong cold stress
PET ≤ −8	Very cold	Extreme cold stress

Source: Reference [50].

**Table 6:** Results of ANOVA on PET in winter and summer.

			BN	BD	BF	VD	VC
F and significance	Winter	8:00	18.508 **	1.561	-	4.366 *	43.680 **
		9:00	4.375 *	1.990	-	15.887 **	-
		10:00	1.691	-	1.694	3.113	12.838 **
		11:00	1.855	3.187	5.985 *	4.445 *	110.578 **
		12:00	1.121	5.477 *	5.816 *	2.548	163.134 **
		13:00	9.449 **	8.851 **	1.399	11.065 **	242.513 **
		14:00	-	4.354 *	2.868	4.004 *	125.705 **
		15:00	1.001	2.316	3.602	1.533	72.973 **
		16:00	1.441	2.597	6.738 *	2.097	78.737 **
		17:00	1.773	-	2.981	4.616 *	20.412 **
		18:00	16.708 **	3.172	-	2.655	58.622 **
		dPET	1.677	3.602	4.041 *	3.669	61.711 **
	Summer	8:00	6.050 *	1.184	9.149 **	16.354 **	6.202 *
		9:00	4.163 *	-	11.164 **	9.090 **	49.144 **
		10:00	8.817 **	-	11.239 **	1.674	64.672 **
		11:00	4.874 *	-	9.289 **	1.187	134.361 **
		12:00	7.785 **	1.329	16.634 **	7.292 **	327.907 **
		13:00	14.235 **	1.330	15.241 **	17.091 **	256.215 **
		14:00	7.803 **	2.159	10.642 **	16.205 **	396.589 **
		15:00	3.882 *	1.712	11.333 **	4.925 *	459.617 **
		16:00	3.320	1.070	7.050 **	1.537	141.514 **
		17:00	3.578	-	8.585 **	5.890 *	79.844 **
		18:00	3.929 *	-	5.176 *	9.773 **	37.038 **
		dPET	6.352 *	1.231	12.357 **	2.401	136.131 **

* F_0.05_ < F < F_0.01_, correlation is significant; ** F > F_0.01_, correlation is highly significant; F_0.05_ = 3.84, F_0.01_ = 7.01.

**Table 7:** The dPET, ΔdPET_W_, ΔdPET_S_, and ΔPET values of each simulation scheme.

Scheme	ΔdPET_W_ (°C)	ΔdPET_S_ (°C)	ΔPET (°C)
S1	6.10	8.77	14.87
S2	6.29	8.15	14.44
S3	6.54	7.42	13.96
S4	6.49	7.22	13.71
S5	6.07	9.04	15.11
S6	6.65	7.42	14.07
S7	5.96	8.96	14.92
S8	6.34	8.31	14.65
S9	6.40	7.64	14.04
S10	6.40	7.78	14.18
S11	6.35	8.00	14.35
S12	6.54	7.09	13.63
S13	6.59	7.00	13.59
S14	5.91	9.11	15.02
S15	5.97	8.55	14.52
S16	6.02	8.69	14.71
S17	6.27	7.78	14.05
S18	6.18	8.06	14.24
S19	6.41	7.70	14.11
S20	6.42	6.95	13.37
S21	6.45	6.91	13.36
S22	6.49	7.24	13.73
S23	6.15	8.62	14.77
S24	6.13	8.25	14.38
S25	6.27	7.66	13.93

**Table 8:** ANOVA results of ΔPET.

	BN	BD	BF	VD	VC
F value	11.266	-	10.744	4.256	96.705
Significance	Highly significant	Not significant	Highly significant	Significant	Highly significant

F_0.05_ = 3.84; F_0.01_ = 7.01.

## Data Availability

The meteorological data entered into the software on the day of measurement came from Huiju Meteorological Data can be downloaded from http://hz.hjhj-e.com/home/meteorologicalData/dataDetails (accessed on 11 July 2021). Data of typical meteorological days for the simulated 25 schemes can be obtained from *Design standard for thermal environment of urban residential areas (JGJ 286-2013)* and exposure draft.

## Appendix A

The appendix contains simulation results (mean PET values of the entire Linpan area) of 25 schemes at different times of the day during winter and summer.

**Table A1:** Hourly average PET-value changes of each scheme in winter and summer.

Season	Scheme	Hourly Average PET Values											
		08:00	09:00	10:00	11:00	12:00	13:00	14:00	15:00	16:00	17:00	18:00	dPET
Winter	S1	2.36	4.58	7.57	9.57	10.94	11.71	12.19	12.19	11.47	9.52	5.84	8.9
	S2	2.42	4.65	7.4	9.34	10.66	11.4	11.81	11.79	11.05	9.33	5.98	8.71
	S3	2.76	4.4	6.89	8.88	10.34	11.18	11.55	11.45	10.62	8.86	6.09	8.46
	S4	2.79	4.76	7.1	8.94	10.2	11.14	11.41	11.33	10.62	9.1	6.25	8.51
	S5	2.18	4.69	7.63	9.64	11.01	11.8	12.22	12.23	11.47	9.62	5.76	8.93
	S6	2.9	4.45	6.76	8.73	10.16	10.98	11.34	11.19	10.42	8.76	6.2	8.35
	S7	2.29	4.69	7.66	9.79	11.05	11.98	12.34	12.37	11.64	9.69	5.9	9.04
	S8	2.44	4.55	7.27	9.26	10.63	11.46	11.77	11.73	11.02	9.27	5.87	8.66
	S9	2.79	4.36	6.96	9.13	10.49	11.52	11.79	11.67	10.9	8.97	6.06	8.6
	S10	2.75	4.56	7.11	9.1	10.49	11.28	11.62	11.55	10.83	9.1	6.18	8.6
	S11	2.73	4.68	7.2	9.15	10.53	11.4	11.67	11.57	10.88	9.2	6.09	8.65
	S12	3.09	4.47	6.75	8.85	10.22	11.26	11.48	11.31	10.55	8.79	6.28	8.46
	S13	2.88	4.69	6.97	8.85	10.11	11.03	11.31	11.21	10.48	8.91	6.22	8.41
	S14	2.39	4.65	7.7	9.81	11.18	11.94	12.43	12.47	11.75	9.7	5.92	9.09
	S15	2.52	4.88	7.67	9.69	10.96	11.85	12.25	12.23	11.53	9.68	6.07	9.03
	S16	2.33	4.72	7.66	9.7	11.06	11.91	12.22	12.2	11.47	9.62	5.84	8.98
	S17	2.89	4.47	6.99	9.28	10.77	11.77	11.97	11.81	10.99	8.97	6.15	8.73
	S18	2.66	4.8	7.43	9.41	10.66	11.53	11.9	11.88	11.21	9.45	6.14	8.82
	S19	2.92	4.69	7.08	9.02	10.42	11.26	11.56	11.48	10.77	9.09	6.18	8.59
	S20	3.06	4.68	7.13	9	10.27	11.22	11.51	11.43	10.77	9.09	6.24	8.58
	S21	2.98	4.83	7.04	8.84	10.28	11.48	11.53	11.28	10.51	8.99	6.28	8.55
	S22	3.17	4.66	6.98	8.89	10.24	11.09	11.34	11.25	10.6	9.02	6.37	8.51
	S23	2.64	4.42	7.2	9.52	10.87	11.84	12.16	12.1	11.36	9.29	5.98	8.85
	S24	2.79	4.49	7.24	9.5	10.95	11.86	12.11	12.01	11.26	9.22	6.12	8.87
	S25	3.02	4.53	7	9.19	10.71	11.7	11.95	11.76	10.91	8.99	6.23	8.73
Summer	S1	23.53	27.38	30.03	31.28	31.4	31.49	32.46	33.56	34.03	33.08	30.24	30.77
	S2	23.45	26.94	29.48	30.56	30.59	30.76	31.61	32.68	33.23	32.43	29.89	30.15
	S3	22.58	25.79	28.72	30.16	30.3	30.69	31.43	32.17	32.3	31.11	28.35	29.42
	S4	23.27	26.12	28.64	29.74	29.66	30.02	30.65	31.4	31.93	31.16	28.83	29.22
	S5	23.83	27.69	30.19	31.46	31.45	31.41	32.52	33.88	34.48	33.59	30.9	31.04
	S6	22.98	26.09	28.81	30.04	30.08	30.46	31.14	31.91	32.23	31.24	28.6	29.42
	S7	23.65	27.74	30.26	31.45	31.37	31.35	32.37	33.83	34.4	33.51	30.59	30.96
	S8	23.28	27	29.51	30.8	30.79	30.87	31.84	33.08	33.57	32.67	29.96	30.31
	S9	22.45	26.01	28.9	30.39	30.49	30.8	31.66	32.54	32.74	31.46	28.57	29.64
	S10	23.35	26.59	29.21	30.32	30.25	30.54	31.25	32.16	32.72	31.91	29.29	29.78
	S11	23.29	26.82	29.27	30.5	30.51	30.67	31.54	32.59	33.05	32.23	29.58	30
	S12	22.42	25.74	28.44	29.8	29.85	30.2	30.93	31.67	31.94	30.84	28.13	29.09
	S13	22.92	25.85	28.33	29.44	29.41	29.73	30.49	31.33	31.83	31.02	28.64	29
	S14	23.39	27.57	30.32	31.71	31.77	31.73	32.84	34.21	34.64	33.55	30.44	31.11
	S15	23.62	27.37	29.84	31.01	31	31.06	32.06	33.27	33.77	32.9	30.14	30.55
	S16	23.35	27.33	29.78	31.1	31.17	31.07	32.27	33.68	34.18	33.23	30.48	30.69
	S17	22.26	25.89	28.9	30.62	30.78	30.97	32	33.02	33.05	31.54	28.6	29.78
	S18	23.55	26.95	29.36	30.49	30.49	30.59	31.48	32.6	33.08	32.32	29.73	30.06
	S19	23.35	26.61	29.03	30.16	30.08	30.3	31.09	32.1	32.67	31.93	29.39	29.7
	S20	22.72	25.73	28.27	29.49	29.54	29.93	30.61	31.35	31.72	30.74	28.32	28.95
	S21	22.62	25.56	27.91	29.38	29.61	29.96	30.75	31.56	31.62	30.66	28.34	28.91
	S22	23.26	26.19	28.62	29.73	29.69	30.01	30.64	31.46	31.96	31.25	28.85	29.24
	S23	22.75	27	29.79	31.36	31.56	31.47	32.58	33.9	34.07	32.83	29.54	30.62
	S24	22.91	26.97	29.6	30.86	30.83	30.84	31.88	33.11	33.6	32.59	29.55	30.25
	S25	22.48	25.93	28.82	30.37	30.53	30.75	31.7	32.68	32.83	31.51	28.64	29.66

dPET, average value of results from 08:00 to 18:00.
